# GOPred: GO Molecular Function Prediction by Combined Classifiers

**DOI:** 10.1371/journal.pone.0012382

**Published:** 2010-08-31

**Authors:** Ömer Sinan Saraç, Volkan Atalay, Rengul Cetin-Atalay

**Affiliations:** 1 Department of Computer Engineering, Middle East Technical University, Ankara, Turkey; 2 Department of Molecular Biology and Genetics, Faculty of Science, Bilkent University, Ankara, Turkey; University College Dublin, Ireland

## Abstract

Functional protein annotation is an important matter for *in vivo* and *in silico* biology. Several computational methods have been proposed that make use of a wide range of features such as motifs, domains, homology, structure and physicochemical properties. There is no single method that performs best in all functional classification problems because information obtained using any of these features depends on the function to be assigned to the protein. In this study, we portray a novel approach that combines different methods to better represent protein function. First, we formulated the function annotation problem as a classification problem defined on 300 different Gene Ontology (GO) terms from molecular function aspect. We presented a method to form positive and negative training examples while taking into account the directed acyclic graph (DAG) structure and evidence codes of GO. We applied three different methods and their combinations. Results show that combining different methods improves prediction accuracy in most cases. The proposed method, GOPred, is available as an online computational annotation tool (http://kinaz.fen.bilkent.edu.tr/gopred).

## Introduction

Due to advances in genome sequencing techniques during the last decade, the number of proteins being identified is exponentially increasing. Functional annotation of proteins has become one of the central problems in molecular biology. Manually curating annotations turns out to be impossible because of the large amount of data. Thus, computational methods are becoming important to assist the biologist in this tedious work.

Attempts to automate function annotation follow two main tracks in the literature. In the first track, the protein to be annotated is searched against public databases of already annotated proteins. Annotations of the highest-scoring hits, according to a similarity calculation, are transfered onto the target protein. This track can be called the *transfer approach*. Despite some known drawbacks such as excessive transfering of annotations, low sensitivity, low specificity, and propagation of database errors, this track is the most widely used among biologists because as it is historically the first successful method but developed when the number of protein sequences in the databases was much lower than today's [1]–[6], it is well understood and widely used by the experimentalists.

In the second track, protein annotation is formulated as a classification problem where annotations are classes and proteins are samples to be classified. This so-called *classification approach* is based on sophisticated and powerful classification algorithms such as support vector machines (SVMs) and artificial neural networks (ANNs) [7]. Methods following the classification approach explicitly draw a boundary between proteins, negative and positive training samples, defined in terms of functional annotation. Since the classification approach considers both negative and positive annotations, such methods have been shown to be more accurate in many cases [8]. Yet, they are not as popular among biologists as one would expect. One reason is because classification approaches require well-defined annotation classes and positive and negative training data for each class. The protein functional annotation task is open to more than one interpretation, where the exact annotation depends on the context in which the protein is used [5]. Furthermore, similar functions can be referred to by annotation terms with different levels of specificity. Thus, to train classifiers, one would first need a controlled vocabulary for functional terms. Then, positive and negative training data must be collected for each of these terms or classes. Data preparation is not straightforward because functional terms are related and proteins may have more than one annotation. We believe that if one can establish a classification framework with a rich number of well-assigned functional annotation terms and high quality training data, methods in classification approach will receive more attention.

In the literature, there is a wide range of methods that follow the classification approach for automated functional annotation in the literature. These methods can be grouped into three categories, depending on the employed features:

1. homology-based methods,
2. subsequence-based methods,
3. feature-based methods.

Homology-based methods use the target protein's overall sequence similarity to positive and negative sequence data in order to decide to which functional class it belongs. It is generally accepted that a high level of sequence similarity is a strong indicator of functional homology. The most well-established and widely used methods for finding sequence similarity are local alignment search tools such as BLAST and PSI-BLAST [9], [10]. Subsequence-based methods focus on highly conserved subregions such as motifs or domains that are critical for a protein to perform a specific function. These methods are especially effective when the annotation to be assigned requires a specific motif or domain. The existence of these highly conserved regions in a protein enables us to infer a specific annotation even in remote homology situations [11]–[18]. In feature-based methods, biologically meaningful properties of a protein such as frequency of residues, molecular weight, secondary structure, extinction coefficients and other physicochemical properties are extracted from the primary sequence. These properties are then arranged as feature vectors and used as input to classification techniques [7], [19]–[24].

Each of the above approaches has different strength and weaknesses in classifying different functional terms. For example, the immunoglobulin's three dimensional structure is a good distinguishing feature, thus a homology-based approach that considers overall sequence similarity would be effective in identifying immunoglobulins. As secreted proteins carry a signal peptide despite their dissimilar amino acid sequence, a subsequence-based approach would be more appealing for recognizing these types proteins. The hydrophobic core is a hallmark of transmembrane proteins hence a method that considers the hydrophobicity of residues is a better classifier of these structures. Because of such characteristics, combining methods from different approaches would be more successful to classify of a wide range of protein functions than using a single method.

Our study applies and investigates the effect of combining different classifiers in order to improve the accuracy of classifying proteins according to their functions. We compare the results of three different annotation methods and four different combinations of these methods. In this study, we developed a method to prepare training data for the terms defined in Gene Ontology (GO) framework. Then, we focused on annotating proteins with 300 GO molecular function (MF) terms. We keep to the molecular function aspect mainly because genes annotated by a MF term are more likely to share a common sequence, subsequence or physicochemical features related to that specific function. Gene Ontology terms for biological process (BP) or cellular component(CC) aspects of GO may include genes with diverse features in the same class and similar features in different classes, thus this pose a problem for the classifier. This problem may not be as severe for homology-based approaches because the decision is made by considering only a few high-scoring hits independent of the other class members. On the other hand, the decision boundary for classes in a discriminative approach is optimized by considering all positive and negative samples. Although it is possible to design classifers that are more appropriate for classifying BP and CC terms, that is outside of the scope of this study.

We formulated the problem as a classification problem with 300 classes, where proteins can be assigned to more than one class. In order to avoid a bias towards a larger negative class, we presented a threshold relaxation method that not only shifts the threshold towards the more appropriate classification boundary but also maps the output of the classifier to a probability value. Finally, we investigated the effect of different classifier combination methods; results showed that combining methods improved performance for about 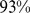 of the classes.

Previously we developed SPMap, which predicts protein function based on subsequence feature space mapping. The difference of this work and the previous SPMap is that SPMap is one of the three employed classifiers. In addition to SPMap, in this work, we have devised and implemented BLAST k-nearest neighbor (BLAST-kNN) and peptide statistics combined with SVMs (PEPSTATS-SVM). To the best of our knowledge, this is the first study to combine multiple classifiers for protein function prediction and this is the most comprehensive discriminative classification approach that covers so many GO terms.

## Materials and Methods

We performed tests for 300 GO terms in a one-versus-all setting. For each GO term, statistics were obtained by the average results from 5-fold cross-validation. In order to calculate the probability described in Section *Threshold Relaxation* and also the ROC scores for weighted mean method, we used leave-one-out cross validation in the test set. In other words, we used all available test dataset but one as the *helper set* and one held-out sample as the *validation set*. This was performed for all of the test datasets.

In order to compare the methods and combination strategies, we made use of 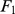 statistics, which are more robust in the case of uneven test sets [25]. When the sizes of positive and negative test sets are unbalanced, several common statistics such as sensitivity, specificity and accuracy may overstate or understate the classification's performance. The 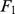 measure is the harmonic mean between precision and recall.
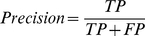
(1)

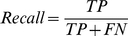
(2)

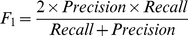
(3)

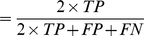
(4)TP, FP, TN, and FN denotes true positive, false positive, true negative and false negative, respectively.

There are more than 8600 GO terms under the *molecular function* aspect and most have very little associated gene products, if any, or they are organism specific. To have enough data to reliably assess performance we only chose GO terms with at least 100 associated gene products. (Note that 100 gene products is not a lower limit for training GO terms.) Also, we removed broad GO terms like *binding* because they are not very informative. The remaining set corresponds to 300 GO terms at the time of implementation. The classifier for each GO term is independent of the rest of the system; more can be added on demand, even for terms with very few gene products.

### Dataset Preparation

One of the most well-known and widely used attempts to standardize protein function terms and to define their relations is Gene Ontology, providing ontology in three aspects: *molecular function*, *biological process* and *cellular location*. In this study, we focused on *molecular function* aspect. GO organizes molecular functions as nodes on a directed acyclic graph (DAG). As a node is a more specific case of its parent node or nodes (a node may have more than one parent), it is critical to select positive and negative annotation data sets. Here, we present a way of establishing positive and negative training data for each class based on evidence codes provided by the GO annotation (GOA) project and by considering the structure of the GO DAG. While preparing training data, we used UniProt release 13.0 as the source for protein sequences [26]. Annotations were obtained from October, 2007 version of GOA mapping file and the October 2007 version of GO ontology is used as the bases of the functional terms and their relations in our system. We give the lists of UniProt identifiers of proteins used as positive and negative samples for 300 GO terms in Supplementary Dataset S1.

#### Positive Training Set

**Figure 1:**
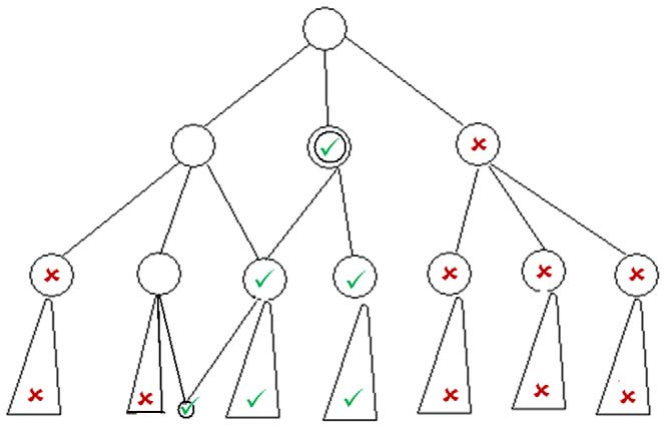
Sample GO DAG showing how we prepared positive and negative training data. The double-circled node indicates the target term. Nodes with green check symbol represent terms included in the positive dataset while those labeled by red X symbol represent terms included in the negative dataset.

Preparing the positive training dataset was relatively simple compared to the negative dataset. First, we extracted all proteins that have been annotated with the target term or one of its descendants that are connected with a *is_a* relation. There are also *part_of* and *controlled_by* relations in GO but for the molecular function aspect, they were negligible. Figure 1 shows this process graphically. In this figure, nodes with a check symbol represent terms included in the positive dataset. In order to populate a training dataset without any bias towards computational prediction methods and to reduce the noise in the training data as much as possible, we filtered out proteins that are annotated with one of IC, IEA, ISS, NAS and ND evidence codes (see Table 1). These codes refer to annotations either obtained by electronic means or have ambiguity in their origin [27]. The remaining evidence codes, IDA, IEP, IGI, IMP, IPI, RCA and TAS refer to experimental evidences that we included in our study.

**Table 1:** Evidence codes used by the GOA Project.

Code	Explanation
IDA	Inferred from Direct Assay
IEP	Inferred from Expression Pattern
IGI	Inferred from Genetic Expression
IMP	Inferred from Mutant Phenotype
IPI	Inferred from Physical Interaction
RCA	Inferred from Reviewed Computational Analysis
TAS	Traceable Author Statement
IC	Inferred by Curator
IEA	Inferred by Electronic Annotation
ISS	Inferred from Sequence or Structural Similarity
NAS	Non-Traceable Author Statement
ND	No biological Data available

#### Negative Training Set

Theoretically, an annotation for a protein only specifies the function it performs. This is generally not an indication of what it does not perform. A protein without a specific functional label might merely be due to lack of evidence experiment. Although this may not be a severe problem in practice, it helps us understand the difficulties in constructing a negative training dataset for a target annotation term. As a result, each protein that does not have an annotation of a target class or one of its descendants is a probable negative training sample. However, including all such proteins in the negative training dataset is neither useful nor necessary. First of all, positive and negative training sets' sizes may become very unbalanced in such a case. For some functional classes, the size of the positive training dataset is in the order of tens of proteins, whereas it is about tens of thousands for the negative dataset. Second, computational cost increases with the size of the training dataset. Since we trained our classifiers in a one-versus-all setting for 300 GO molecular function terms, our strategy was to select random representative sequences (at most 10) from each GO term other than the target term. In Figure 1, nodes with an X symbol represent GO terms that can be included in the negative dataset. We imposed two conditions on the selected random representative sequences:

1. A sequence should not be annotated with the target term or one of its descendant terms.
2. If a sequence is annotated with one of the ancestors of the target term, it should also be annotated with a sibling of the target term.

The first condition is straight-forward because we don't want to include protein sequences that are already in the positive training data. The second constraint is imposed in order to avoid including prospective positive training data into the negative dataset. Ideally, each protein should be annotated with a GO term on a leaf node, in other words, with the most specific annotation. If a protein is annotated only up to an internal node, this means either that there is lack of evidence for a more specific annotation or an appropriate GO term for that protein has not yet been added to the ontology. Thus, we excluded proteins that are annotated by an ancestor GO term but not with a sibling.

We aim to differentiate the proteins annotated with sibling terms; therefore proteins annotated with a sibling term should be in the negative dataset. However, the proteins with shared ancestral GO terms which are not annotated with a sibling GO term are susceptible to be annotated with the current GO term. Hence, we include them neither to the positive dataset nor to the negative dataset.

### Classification Methods

After preparing positive and negative training data for each of 300 GO molecular function terms, we applied three classification methods representing three annotation approaches:

- BLAST *k*-nearest neighbor (BLAST-*k*NN) for homology-based method,
- Subsequence Profile Map (SPMap) for the subsequence-based method,
- Peptide statistics combined with SVMs (PEPSTATS-SVM) for the feature-based method.

#### BLAST-kNN

In order to classify the target protein, we used the 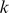-nearest neighbor algorithm [28]. Similarities between the target protein and proteins in the training data were calculated using the NCBI-BLAST tool. We extracted 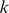-nearest neighbors with the highest 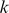 BLAST score. The output of BLAST-kNN, 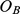 for a target protein, is calculated as follows:
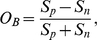
(5)where 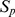 is the sum of BLAST scores of proteins in the 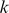-nearest neighbors in the positive training data. Similarly, 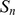 is the sum of scores of the 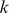-nearest neighbor proteins in the negative training data. Note that the value of 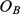 is between −1 and +1. The output is 1 if all 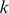 nearest proteins are elements of the positive training dataset and −1 if all 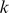 proteins are from the negative training dataset. In order to determine the label, instead of directly using 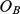 with a fixed threshold, we employed the threshold relaxation algorithm given in the section entitled **Threshold Relaxation**, below.

#### SPMap

SPMap maps protein sequences to a fixed-dimensional feature vector, where each dimension represents a group of similar fixed-length subsequences [18]. Supplementary Figure S1 gives an overview of SPMap. In order to obtain groups of similar subsequences, SPMap first extracts all possible subsequences from the positive training data and clusters similar subsequences. A probabilistic profile or a position-specific scoring matrix is then generated for a cluster. The number of clusters determines the dimension of the feature space. The generation of these profiles constructs the feature space map. Once this map is constructed, it is used to represent protein sequences as fixed dimensional vectors. Each dimension of the feature vector is the probability, calculated by the best matching subsequence of the protein sequence to the corresponding probabilistic profile. If the sequence to be mapped contains a subsequence similar to a specific group, the value of the corresponding dimension will be high. Note that this representation reflects the information of subsequences that are highly conserved among the positive training data. After feature vectors have been constructed, SVMs are used to train classifiers. Further information on SPMap is found in [18].

#### Pepstats-SVM

The *Pepstats* tool which is a part of the European Molecular Biology Open Software Suite (EMBOSS) and used to extract the peptide statistics of the proteins [29]. Each protein is represented by a 37-dimensional vector. Peptide features and their dimensions are given in Table 2. These features are scaled using the ranges of the positive training data for both the training and test datasets and then fed to an SVM classifier.

**Table 2:** Features used in Pepstats-SVM and their dimensions.

Feature	Dimension
Molecular Weight	1
Number of residues	1
Average residues weight	1
Isoelectric point	1
Charge	1
A280 Molar Extinction Coefficient	1
A280 Extinction Coefficient 1mg/ml	1
Improbability of expression in inclusion bodies	1
Dayhoff Statistics for each amino acid	20
Percent of tiny residues	1
Percent of small residues	1
Percent of aliphatic residues	1
Percent of aromatic residues	1
Percent of non-polar residues	1
Percent of polar residues	1
Percent of charged residues	1
Percent of basic residues	1
Percent of acidic residues	1
Total	37

### Threshold Relaxation

A support vector machine finds a separating decision surface (hyperplane) between two classes that maximizes the margin, which is the distance of that hyperplane to the nearest samples. For a new sample, the output of the SVM is the distance of the hyperplane to the new sample. The sign of the output determines on which side of the hyperplane the new sample resides. Hence, the natural threshold for SVM is zero. The optimization algorithm of SVM that finds the hyperplane maximizing the margin is data-driven and may be biased towards the classes with more training samples. Therefore, using the natural threshold usually results in poor sensitivity if the sizes of the positive and negative training datasets are unbalanced. This is exactly the case in our problem. There are studies in the literature about threshold relaxation in favor of the smaller class [30]–[32]. In our study, we present a method that implicitly adjusts the threshold value and at the same time defines a probability 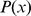 of a sample 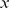 to be in the positive class.

First, we split the test data into two sets, a *helper set*, to calculate the probability 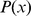, and a held-out *validation set* to evaluate the performance of the method. Since, the number of positive test samples is outnumbered by the negative test samples, our method should handle this unbalanced situation. We calculated a confidence value for the new sample to be positive and negative separately and we then combined these confidences into a single probability. The confidence for the new sample for being positive 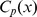, is calculated as the ratio of the number of positive samples in helper set having a classifier output lower than that of the new sample to the number of all positive samples in the helper set. The confidence for being negative, 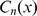, is calculated similarly (Equation 6 and Equation 7). These two ratios are combined to calculate the probability of the new sample being in the positive class (Equation 8). A new sample is predicted to be positive if 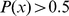, and to be negative, otherwise.
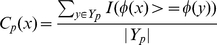
(6)

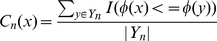
(7)

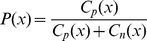
(8)

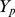
 and 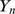 are the positive and negative test samples in the helper set, respectively. 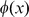 denotes the output of the classifier for sample 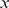. 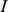 operator returns 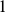 if the condition holds, 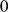 otherwise. 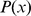 for the classifier output 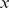 approaches 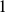 if the fraction of the positive helper test set with classifier output values smaller than 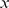 increases or the fraction of the negative helper test set with classifier output values larger than 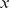 decreases. Note that this method implicitly adjusts the threshold because natural threshold 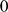 does not necessarily corresponds to a 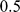 value for 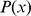. This is clearly observed when we draw the distribution of the elements of positive and negative test data sets with respect to the confidence values as shown in Supplementary Material S1. Furthermore, confidence value provides the user a measure for assessing how probable it is that the sample is a member of the given class.

It is important to note that this confidence value is not assessing the quality of the prediction. It just indicates how far the prediction value of the instance, from the decision boundary learned by the classifier. It doesn't say anything about the quality of the decision boundary, hence the accuracy of the overall classification. The confidence value of the classification is calculated for a single sample using the helper set. On the other hand, the overall accuracy is calculated using all of the samples in the validation test set.

### Classifier Combination

Observations of many classification problems with different classification methods have shown that although there is usually a best method for a specific problem, samples that are correctly classified or misclassified by different methods may not necessarily overlap [33]. This observation led to the idea of combining classifiers in order to achieve a greater accuracy [33], [34]. In this study, we investigated four classifier combination techniques,

1. voting,
2. mean,
3. weighted mean and
4. addition

for three different classification methods.

*Voting*, also known as majority voting, simply decides the class of the new sample by counting positive and negative votes from each classifier. Note that each vote has equal weight and the output values of the classifiers are not taken into account.

For the *Mean* combination method, the mean of the probability values calculated by Equation 8 is used to decide the class of the new sample. If this mean value is greater than 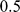, the sample is labeled as positive.

The combination method *Mean* treats each method equally. But the performances of the methods vary for different functional classes. Thus in the *weighted mean* method, we assigned weights to each method depending on their performance in the functional class for which the classifier combination is used. To assess the performance of the methods we made use of the area under the Receiver Operating Characteristic (ROC) curve, which is called the ROC score and widely used measure to evaluate the performance of classification methods. The ROC score estimates the discriminative power of the method independent of the threshold value. To calculate the ROC score of each method, we used the helper test sets. Recall that helper test sets are held out subsets from the test set. To avoid bias, we did not use them in training or performance evaluation. They are only used to calculate ROC scores to calculate weights and for threshold relaxation. We assigned a weight to each method calculated by Equation 9.
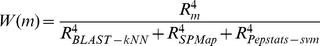
(9)

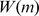
 denotes the weight of method 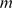, where 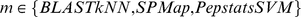. 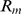 is the ROC score for method 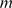. Note that we used the 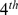 power of ROC scores to assign a higher weight to the method with a better ROC score.

In the *Addition* method, the output values of the classification methods are added directly. The probability defined in Equation 8 is then calculated using these added values.

## Results and Discussion

**Figure 2:**
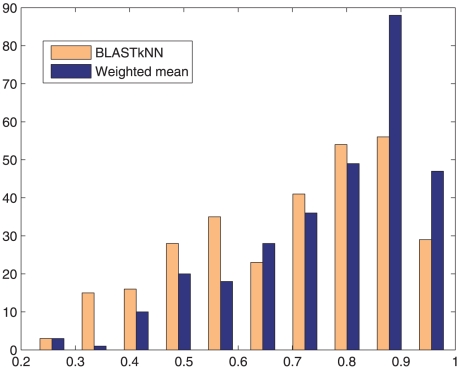
Histogram of 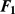 scores of BLAST-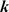
NN and Weighted Mean methods for 300 GO terms.

The *Weighted mean* method performed best in 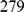 of 300 classifiers, with an average 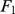 score of 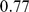. Thus, *Weighted mean* method is chosen as the basis combination method for our online tool *GOPred*. *Addition* was the best for eight classes. *Voting* and *mean* were the best methods for one and 3 of the classes, respectively. Overall, combining improved the performance of 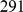 of 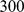 classes. One should note that for the rest of the cases, at least one combination method performed very similar to the best-performing single method. Average sensitivity, specificity and 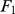 scores over 300 classes are given in Table 3. With respect to 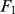 scores, as BLAST-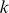
NN and weighted mean methods are the best-performing single and combination methods, respectively, we compared these two methods in order to justify the significance of the improvement obtained by combined classifiers. The histogram of 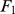 scores of BLAST-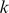
NN and weighted mean methods for 300 GO terms are shown in Figure 2. It can be seen that the distributions are not normal. Hence, instead of the Student's t-test, we used the *Wilcoxon signed-rank* test, which has no normality assumptions [35]. The null hypothesis which states that the means are the same, is rejected with 1% significance level. This justifies that weighted mean performs significantly better than BLAST-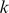
NN.

**Table 3:** Average 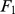 scores, sensitivity and specificity values over 300 GO functional term classifiers.

Method	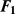	Sensitivity	Specificity	Precision
SPMap	0.62	89.12	88.92	0.51
BLAST-*k*NN	0.70	92.07	92.53	0.59
Pepstats-SVM	0.39	75.47	75.48	0.29
Voting	0.71	90.50	92.85	0.61
Mean	0.74	91.11	93.74	0.65
Weighted Mean	0.77	91.82	94.79	0.68
Addition	0.70	92.72	92.49	0.60

With respect to 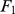 scores, BLAST-kNN turned out to be the best single method for a majority of the functional terms, while outperformed by SPMap only in a small fraction of functional terms. Pepstats-SVM gave the least satisfactory results in all functional classes. Our results indicated that simple peptide statistics were not sufficient to accurately classify GO functional terms. Nevertheless, samples correctly classified by each of the three methods did not overlap; this explains the success of the combination methods. We clearly demonstrate that combining three methods gives the best accuracy for functionally annotating protein sequences.

**Figure 3:**
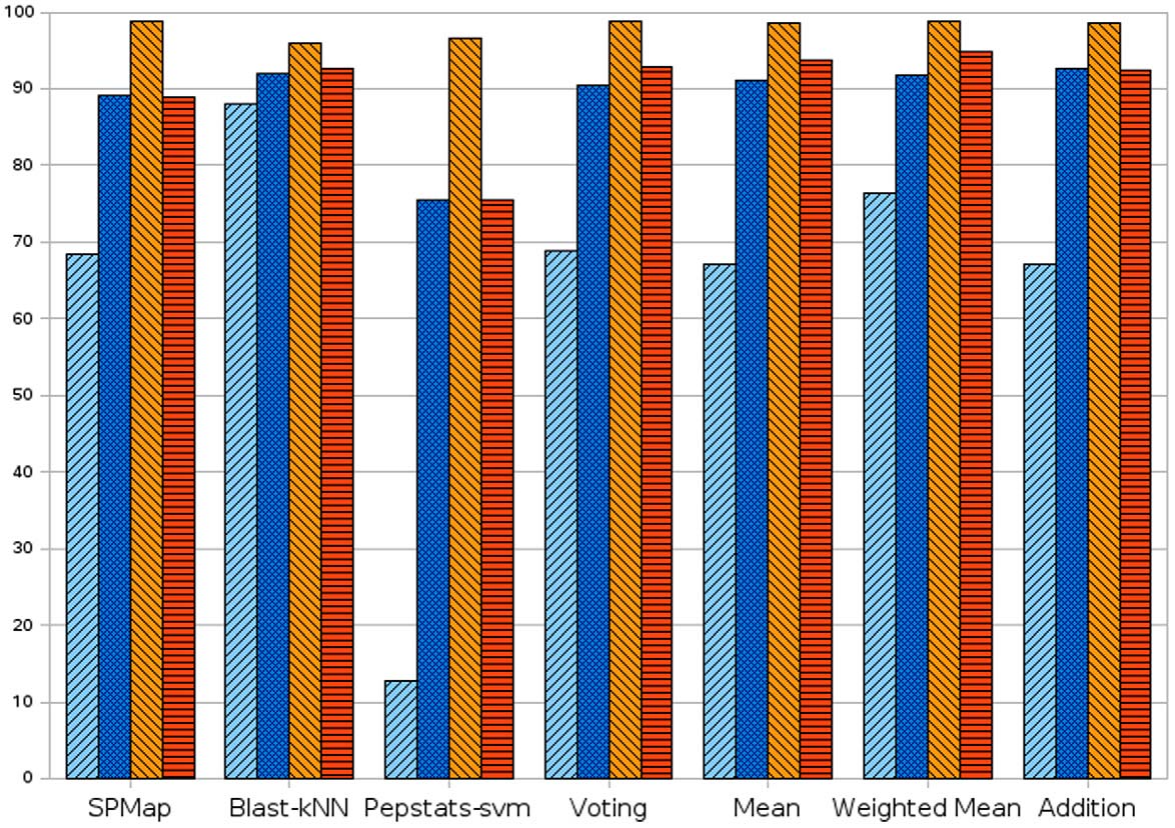
Comparison of sensitivity and specificity values with and without threshold relaxation. The first and second columns are the sensitivity without and with threshold relaxation, third and forth columns are the specificity without and with threshold relaxation.

In order to investigate the effect of the *threshold relaxation* method, we repeated the whole experiment by using natural threshold 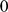 for all methods. Figure 3 shows the comparison of average sensitivity and specificity values with and without threshold relaxation over 300 GO terms; Table 4 shows the change in sensitivity, specificity and also the total change. Results using Pepstats-SVM are significantly improved after threshold relaxation. The accuracy of the BLAST-kNN method was not notably affected; this is not surprising since 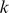-nearest neighbors method does not generate a single decision boundary. After threshold relaxation, there was a small decrease in specificity, but a much larger increase in sensitivity. This confirmed our expectation that there would be a bias towards the class with more training samples. In the majority of the 300 GO terms, the positive training dataset was highly outnumbered by the negative training dataset. Thus, samples tended to be classified as negative. This explains the very high specificity and low sensitivity values when threshold relaxation was not used. Automated function prediction tools are generally used to determine a rough idea about a protein's possible functions before conducting further *in vitro* experiments. We believe that failing to detect an important annotation would have far more severe consequences than assigning a wrong annotation. Thus, increasing sensitivity without a detrimental effect to specificity is a very important achievement. Detailed statistics (dataset sizes, true positive (TP), false positive (FP), true negative (TN), false negative (FN), sensitivity, specificity, positive predictive value (PPV), receiver operating characteristic (ROC) score, 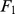 score) for all of the methods on each GO functional term can be found in the Supplementary Material S2.

**Table 4:** Changes in sensitivity and specificity, total change and change in F1 score when threshold relaxation is applied.

Methods	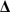 Sensitivity	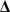 Specificity	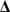 Total	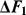
SPMap	20.80	−9.94	10.86	0.14
BLAST-kNN	4.13	−3.44	0.69	−0.10
Pepstats-SVM	62.66	−21.17	41.49	0.26
Voting	21.68	−5.86	15.82	−0.14
Mean	24.02	−4.95	19.07	−0.13
Weighted Mean	15.56	−3.96	11.60	−0.14
Addition	25.63	−6.19	19.44	−0.05

A positive value indicates an increase whereas a negative value indicates a decrease.

The actual challenge for an automated annotation tool is to annotate newly identified sequences or genomes in addition to the validation of the tool on the well established annotations of highly studied proteins. Thus, we applied our method to predict functions of nine recently reported H. sapiens proteins in the last year and highly studied human glucokinase, p53 tumor suppressor, and ras oncogene from NCBI database (Table 5, first 3 columns). For all of the analyzed protein sequences, GOPred was able to predict the literature reported functions of these proteins. This test was a decent indication of the effectiveness of the combination method. Another challenge is the comparison between the performances of the new and the previously reported annotation tools. Currently to the best of our knowledge, there are not any other discriminative classifier approach that performs predictions on GO terms, therefore, we compared GOPred annotations with ConFunc [36], PFP [37], and GOtcha [38] annotations on the above-mentioned twelve protein sequences.

**Table 5:** GOPred, ConFunc, PFP and GOtcha annotations for 12 human gene entries from the NCBI gene database.

Gene Symbol	Literature Report	GOPred annotations:Probability	ConFunc (GO c-value) [36]	PFP: Probabality [37]	GOtcha: Est. likelyhood% [38]
DDX11L1	a protein from novel transcript family from human subtelomeric regions with unestablished function [39]	hydrolase activity, acting on ester bonds: **0.87** protein complex binding: **0.84**	RNA binding (c:4.5569e-05), nucleic acid binding (c:0.00020006), binding (c:0.00020006)	hydrolase activity, acting on acid anhydrides:100%, purine nucleotide binding:100%, binding:98%	catalytic activity:52%, DNA helicase activity:52%, hydrolase activity:52%, helicase activity:52%, nucleoside-triphosphatase activity:52%
KILLIN	Nuclear inhibitor of DNA synthesis with high affinity DNA binding [40]	Exonuclease activity: **0.95**	No results generated because insufficient Annotated sequences were identified	DNA binding:34%, nucleotide binding:26%, ATP binding:26%	Molecular function child node absent
GLRX	glutaredoxin-like, oxidoreductase [41]	oxidoreductase activity: **0.97**	glutathione disulfide oxidoreductase activity (c:1.0138e-08), peptide disulfide oxidoreductase activity (c:1.0138e-08), disulfide oxidoreductase activity (c:7.3644e-08), oxidoreductase activity (c:1.0175e-07), catalytic activity (c:1.0175e-07)	purine nucleotide binding:97%, porter activity:96%, binding:89%, steroid sulfotransferase activity:87%	Molecular function child node absent
FINP2	AMPK and FLCN interaction ([42])	enzyme activator activity: **0.61**, enzyme binding: **0.71**	No results generated because insufficient Annotated sequences were identified	binding:88%, transition metal ion binding:80%, cation binding:71%	Molecular function child node absent
KIF18B	microtubule associated motor protein that use ATP [43]	microtubule binding: **0.88**, motor activity: **0.83**	motor activity (c:1.3769e-17)	purine nucleotide binding:97%, porter activity:96%, binding:89%, steroid sulfotransferase activity:87%	binding:33%, ribonucleotide binding:33%, nucleotide binding:33%, purine nucleotide binding:33%, purine ribonucleotide binding:33%
HELT	transcription regulator activity [44]	protein homodimerization activity: **0.98**, transcription corepressor activity: **0.95**	DNA binding (c:1.2677e-09), nucleic acid binding (c:1.2677e-09), binding (c:1.2677e-09)	hydrolase activity, acting on acid anhydrides:100%, purine nucleotide binding:100%, binding:98%	transcription regulator activity:23%, binding:23%, DNA binding:23%, nucleic acid binding:23%, transcription factor activity:23%
RGL4	guanin nucleotide dissociation [45]	guanyl-nucleotide exchange factor: **0.79**, small GTPase binding: **0.73**	receptor binding (c:1.3056e-10), protein binding (c:2.4304e-09), binding (c:2.4283e-09), Molecular Function (c:4.5798e-10)	binding:78%, cation binding:71%, trimethylamine-N-oxide reductase (cytochrome c) activity:65%, nucleic acid binding:63%	Molecular function child node absent
PGAP1	GPI inositol-deacylase [46]	lipase activity: **0.89**, hydrolase activity acting on ester bonds: **0.89**, acyltransferase activity: **0.79**	phosphoric ester hydrolase activity (c:0), nuclease activity (c:0), hydrolase activity, acting on ester bonds (c:3.0683e-17), hydrolase activity (c:1.5396e-17), catalytic activity (c:1.5396e-17)	cation binding:62%, binding:59%, ion binding:58%, metal ion binding:52%	Molecular function child node absent
COBRA1	member of negative elongation factor complex during transcription, inhibitor of AP1 [47]	ribonucleotide binding: **0.91**, enzyme regulator activity: **0.81**	binding (c:3.361e-18)	binding:88%, transition metal ion binding:80%, cation binding:71%, nucleic acid binding:68%	Molecular function child node absent
GCK	phosphorylation of glucose during glycolysis [48]	carbohydrate kinase activity: **0.98**, ribonucleotide binding: **0.94**, purine nucleotide binding:**0.93**	glucose binding (c:2.7105e-19), monosaccharide binding (c:2.7105e-19), sugar binding (c:2.7105e-19), carbohydrate binding (c:2.7105e-19), binding (c:2.7555e-14)	hexokinase activity:100%, binding:97%, transferase activity, transferring phosphorus-containing groups:89%, catalytic activity:80%, nucleotide binding:77%, glucokinase activity:76%	binding:38%, nucleotide binding:38%, adenyl ribonucleotide binding:38%, ribonucleotide binding:38%, purine nucleotide binding:38%, ATP binding:38%
TP53	p53 tumor supressor, transcription regulation [49]	chromatin binding: **0.97**, protein heterodimerization activity: **0.97**,transition metal ion binding: **0.95**, double-stranded DNA binding: **0.95**, protein dimerization activity: **0.95**, transcription factor activity: **0.95**, zinc ion binding: **0.95**	transcription factor activity (c:3.0644e-12), DNA binding (c:1.0205e-11), nucleic acid binding (c:1.0205e-11) binding (c:1.0205e-11), transcription regulator activity (c:1.1331e-11)	purine nucleotide binding:100%, DNA strand annealing activity:100%, binding:99%, nucleic acid binding:98%, transcription factor activity:94%, single-stranded DNA binding:90%	binding:33%, ion binding:33%, metal ion binding:33%, cation binding:33%, zinc ion binding:33%, transition metal ion binding:33%
HRAS	v-Ha-ras Harvey rat sarcoma viral oncogene homolog [50]	protein C-terminus binding: **0.97**, GTPase activity: **0.96**, ribonucleotide binding: **0.95**, purine ribonucleotide binding: **0.95**, pyrophosphatase activity: **0.93**, guanyl nucleotide binding: **0.90**	GTP-dependent protein binding (c:2.1523e-09), protein binding (c:7.4218e-09), binding (c:1.003e-06)	hydrolase activity, acting on acid anhydrides:100%, purine nucleotide binding:100%, guanyl nucleotide binding:100%, GTP binding:99%, binding:99%	binding:34%, nucleotide binding:34%, purine nucleotide binding:34%, ribonucleotide binding:34%, purine ribonucleotide binding:34%, guanyl ribonucleotide binding:31%, GTP binding:31%

Both GOtcha and PFP improves the simple homology-based approach. PFP takes into account the DAG structure of GO and ranks probable GO terms according to both their frequency of association to similar sequences and the degree of similarity those sequences share with the query. GOtcha calculates term-specific probability (P-score) measures of confidence instead of directly transferring annotations from highest scoring hits. ConFunc generates position specific scoring matrices (PSSMs) for each GO term using the conserved residues among the sequences annotated by the GO term.

**Figure 4:**
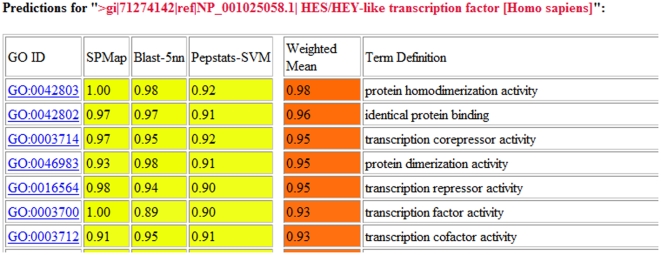
GOPred output for *helt* (HES/HEY-like transcription factor) protein.

DDX11L1 is a novel gene product whose function has not been established yet and it is from human subtelomeric chromosomal region [39]. All of the prediction tools assigned enzyme activity to this protein in relation to nucleic acid chain hydrolysis such as hydrolase activity, acting on ester bonds, nucleic acid binding, acting on acid anhydrides, purine nucleotide binding, and helicase activity. Recently found Killin protein was reported as nuclear inhibitor of DNA synthesis with high DNA binding affinity [40]. For Killin protein, only GOPred and PFP tools generated annotations. GOPred assigned exonuclease activity while PFP gave DNA and nucleotide binding annotations to Killin. Exonucleases are the enzymes that cleave phosphodiester bonds by binding to the DNA; by this way, they may contribute to the nuclear inhibition of DNA synthesis. Another novel protein, GLRX was reported to be glutaredoxin-like, oxidoreductase [41]. All of the tools except GOtcha predicted in general oxidoreductase enzyme activity for GLRX. FINP2 was reported to be interacting partner of AMPK and FLCN proteins [42]. Only GOPred and PFP tools gave predictions in correlation with the function reported in the literature, which were enzyme activator activity, enzyme binding, and purine nucleotide binding. Microtubule associated motor protein KIF18B [43] was predicted as microtubule binding by GOPred and motor activity by both GOPred and ConFunc tools. PFP and GOtcha tools assigned relatively general GO annotations such as hydrolase activity, purine nucleotide binding, and binding. HES-HEY-like transcription factor HELT protein that we also present as an example in Figure 4, has transcription regulator activity [44]. GOPred, ConFunc, and GOtcha prediction tools attributed annotations related to transcription regulation and DNA binding annotations. Recently reported RGL4 protein is a guanine nucleotide dissociation factor [45]. Only GOPred was able to give annotations for RGL4 such as guanyl-nucleotide exchange factor, small GTPase binding that were similar to those reported in the literature. Other annotation tools assigned very general GO terms to RGL4. PGAP1 was reported as GPI inositol-deacylase [46]. GOPred and ConFunc assigned annotations related to the literature reports such as hydrolase activity acting on ester bonds. COBRA1 was the last protein that we included in our analysis as a recently identified protein which was reported as the member of negative elongation factor complex during transcription and inhibitor of AP1 [47]. None of the predictors assigned significant GO terms to COBRA1; some very broad terms such as ribonucleotide binding, nucleic acid binding were predicted.

In addition to the above discussed nine newly identified protein sequences, we analyzed three well characterized proteins. Glucokinase (GCK) is an enzyme that phosphorylates glucose during glycolysis [48]. All of the tools assigned highly significant GO terms related to the function of this protein. p53 tumor suppressor protein (TP53) which is a transcription factor binds to DNA upon tetramerization [49]. GO terms associated to TP53 protein were chromatin binding, protein heterodimerization activity, transcription factor activity, zinc ion binding, DNA binding that were predicted by all of the tools. The oncogene protein v-Ha-ras Harvey rat sarcoma viral oncogene homolog (HRAS) [50] has GTPase activity, which was correctly annotated by all of the tools as well.

The prediction results were very similar for the well-annotated proteins presented in the last three rows of Table 5. GOPred and PFP tools could predict annotations that correlated with the literature reports. However ConFunc did not produce annotations for the protein sequences KILLIN and FINP2. GOthca tool could only assign annotations to the three out of nine newly identified human proteins (Table 5 last column). The comparison here, of course, does not rank the tools' prediction rates, but it gives an idea about their capabilities. The difference observed in comparative function prediction analysis might be due to the underlying methods for these four tools. GOPred and PFP tools apply integration of different data sources related to the sequence to be annotated, rather than searching strict pattern matching to identify functional motifs in the sequences of proteins.

Figure 4 shows the output of our online classification tool for the *helt* protein. Furthermore, as an exemplary genome annotation, GOPred was applied to the annotation of 73 recently reported genes from the Ovis Aries (sheep) genome. Results are available as Supplementary Material S3 and on the GOPred web site (http://kinaz.fen.bilkent.edu.tr/gopred/ovisaries.html).

Automating protein functional annotation is an important and difficult problem in computational biology. Most of the function prediction tools run stand alone and other than those using the *transfer* approach, define the annotation problem as a classification problem. Combining classifiers was shown to improve the accuracy as well as the coverage in protein structure prediction studies [51]. [52] describes the hierarchical composition of two classifiers: a simple classifier with high coverage and another classifier with less coverage but higher accuracy. In contrast, our combination scheme takes into account the results of all classifiers at the same time; it can be thought of as combining evidence from different sources. In addition, we apply it to the totally different context of protein function prediction. Function prediction tools require positive and negative training data and the success of the resulting classifier relies on the representative power of this dataset. In this study, we presented and applied a method to construct well-aimed positive and negative training data using the DAG structure of GO and annotations using evidence codes provided by the GOA project. When using functional classifiers as an annotation system, one must implement a classifier for each functional class in a one-versus-rest setting because as the number of functions increases it becomes intractable to train one-versus-one classifiers. However, a one-versus-rest setting in a classifier renders positive and negative samples highly unbalanced. Therefore, we applied a threshold relaxation method that not only avoids the bias towards the class with more training data but also assigns a probability to the prediction, thus providing a way to assess the strength of the annotation.

There is a rich literature on automated function prediction methods, each of which has different strengths and weaknesses. We investigated the effects of combining different classifiers to better annotate protein sequences with functional terms defined in the molecular function aspect of GO. The resulting combined classifier clearly outperformed constituent classifiers. Our results also showed that the best combination strategy is the *weighted mean* method, which assigns different weights to classifiers depending on their discriminative strengths for a specific functional term.

It is also important to note that we do not merely give annotations but also provide a measure for each functional class that states how probable it is that the query protein is a member of that class. This means we also provide less-probable functional annotations for the analyzed sequence. This information may help the biologist build a road map before conducting expensive *in vitro* experiments.

A valuable addition to GOPred would be to identify important subsequences or physicochemical properties that explains the decisions of GOPred. Unfortunately, a direct interpretation of important features is not possible since the decision boundry for the classification is determined by the non-linear classifier by using the existence and non-existence of features from both positive and negative examples. Furthermore, GOpred is an ensemble of different classifiers. A future work would be to study each classifier separately by feature selection methods and giving probable explanations for each decision.

Finally, the proposed classifier combination approach was made publicly available as an online annotation system, called *GOPred*, covering 300 GO terms. As the classifier for each GO term was trained in a one-versus-rest manner independent of other terms, *GOPred* can be easily extended to cover annotations for more GO terms.

## Supporting Information

Figure S1 Overview of SPMap.
(0.04 MB PDF)

Dataset S1 Dataset: Lists of UniProt IDs of proteins used as positive and negative samples for 300 GO terms.
(14.59 MB TAR)

Material S1 Detailed statistics of test results for 5-fold cross validation on 300 GO terms is available in tab-delimited text format.
(0.16 MB TXT)
