# Supplementary figures and images for: GOPred: GO Molecular Function Prediction by Combined Classifiers
Source: PLoS One. 2010 Aug 31;5(8):e12382. doi: 10.1371/journal.pone.0012382 (PMC2930845; doi:10.1371/journal.pone.0012382)

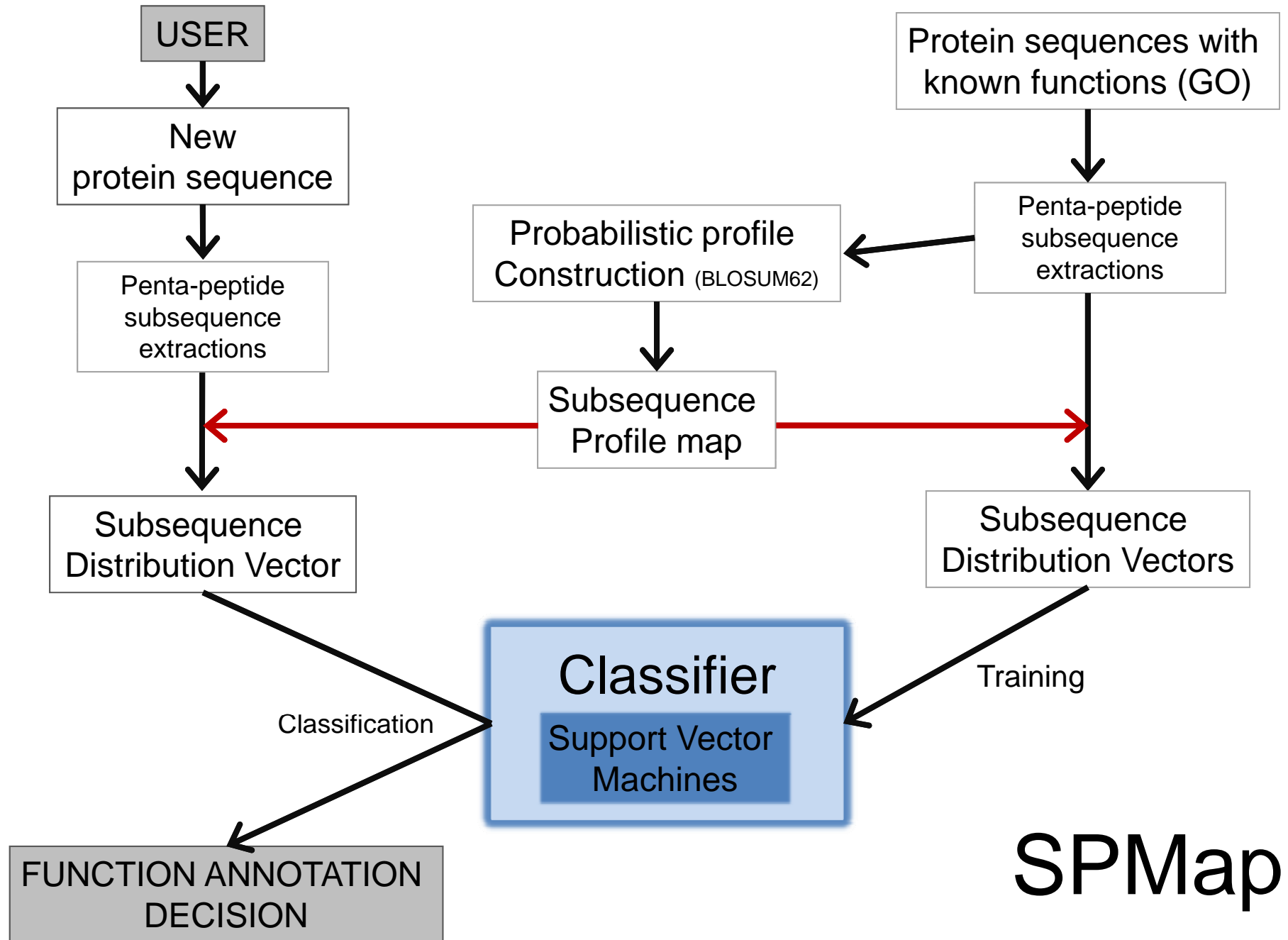

Supplement: Figure S1 — Overview of SPMap. (0.04 MB PDF) [file pone.0012382.s001.pdf]
